# Two new species of *Sugitazyma* (Trimorphomycetaceae, Tremellales) from China: *S. polliae* and *S. pingtangensis*

**DOI:** 10.3897/mycokeys.130.182019

**Published:** 2026-03-27

**Authors:** Peng Wang, Chun-Yue Chai, Qiu-Hong Niu, Feng-Li Hui

**Affiliations:** 1 School of Life Science, Nanyang Normal University, Nanyang 473061, China School of Life Science, Nanyang Normal University Nanyang China; 2 Research Center of Henan Provincial Agricultural Biomass Resource Engineering and Technology, Nanyang Normal University, Nanyang 473061, China Research Center of Henan Provincial Agricultural Biomass Resource Engineering and Technology, Nanyang Normal University Nanyang China

**Keywords:** Basidiomycetous yeast, fungal diversity, phylogenetic analysis, plant, taxonomy

## Abstract

*Sugitazyma* is a genus of basidiomycetous yeasts with only one known species, *Sugitazyma miyagiana*. To further explore the diversity of this genus, field surveys were conducted in Guizhou and Hainan Provinces, China. Phylogenetic analyses based on the internal transcribed spacer (ITS) region and the D1/D2 domain of the large subunit (LSU) rRNA gene revealed that four isolates from plant leaves, represent two new species of *Sugitazyma*: *Sugitazyma polliae***sp. nov**. (holotype strain GDMCC 2.527^T^) and *Sugitazyma pingtangensis***sp. nov**. (holotype strain CICC 33642^T^). Descriptions and illustrations of the two new species are provided, together with comparisons with closely related taxa. A key to the species of *Sugitazyma* is also presented.

## Introduction

The genus *Sugitazyma* was established by Liu et al. (2015) in order to include *S. miyagiana* (Nakase, M. Ito, M. Takem. & Bandoni) Xin Zhan Liu, F.Y. Bai, M. Groenew. & Boekhout (= *Bullera miyagiana* Nakase, M. Ito, Takem. & Bandoni), the only species described so far. It is characterized by its asexual reproduction through polar budding and the formation of ballistoconidia. Physiologically, *S. miyagiana* lacks fermentative ability and is capable of assimilating a variety of carbon sources; however, it fails to assimilate inulin, L-sorbose, methanol, glycerol, and galactitol (Boekhout et al. 2011; Liu et al. 2015).

*Bullera miyagiana* Nakase, M. Ito, Takem. & Bandoni was described by Nakase et al. (1990) based on its phenotypic characteristics, a ballistoconidium-forming yeast. Early phylogenetic analysis of the D1/D2 domain of the LSU rRNA suggested that *B. miyagiana* is a sister species to *B. variabilis* Nakase & M. Suzuki (Fell et al. 2000). However, ITS analysis placed these two species significantly distant from each other (Scorzetti et al. 2002). In a subsequent LSU analysis, the species clustered within the unsupported *Bulleribasidium* clade, indicating that the definitive phylogenetic position of this species remains unresolved (Boekhout et al. 2011). Liu et al. (2015) reconstructed the phylogeny of most described tremellomycetous yeasts and related filamentous fungi using multi-gene sequence analysis. In this study, *B. miyagiana* was positioned on a highly divergent branch, distant from other *Bullera* species, suggesting that it represents an independent lineage within the family Trimorphomycetaceae. As a result, a new genus, *Sugitazyma*, was established for this single-species lineage, thereby restricting the genus *Bullera* to its type lineage in the family Bulleraceae.

The family Trimorphomycetaceae currently comprises four genera, namely *Trimorphomyces*, *Carlosrosaea*, *Saitozyma*, and *Sugitazyma* (Liu et al. 2015). Among them, *Trimorphomyces papilionaceus* Bandoni & Oberw. is the only known species capable of forming sexual morph, in which it parasitizes *Arthrinium sphaerospermum* and produces stalked basidia with cruciate septa (Oberwinkler and Bandoni 1983). The genera in Trimorphomycetaceae are morphologically similar, differing mainly in their physiological and biochemical characteristics. Physiologically, *Sugitazyma* differs from *Trimorphomyces* in its inability to assimilate D-glucosamine, from *Saitozyma* in its ability to assimilate ribitol, and from *Carlosrosaea* in its ability to assimilate erythritol.

*Sugitazyma miyagiana*, has only been reported from Japan (Nakase et al. 1990; Nakase 2000). During our studies of basidiomycetous yeasts inhabiting the surface of plant leaves, we isolated a substantial number of yeasts from various regions in China. Among these isolates, we focus here on four strains of two asexual basidiomycetous yeast species collected from Guizhou and Hainan Provinces, China. Phylogenetic analysis and phenotypic characterization revealed that the isolates represent two new species. The goal of this paper is to describe these two additional species of *Sugitazyma*, which represent the first members of the genus reported from China.

## Materials and methods

### Sample collection and yeast isolation

Fresh leaf samples were collected from Guizhou and Hainan Provinces, China. Each sample was placed in a sterile plastic bag, kept on ice, and transported to the laboratory within 24 hours for sample preparation and subsequent yeast isolation. Yeast strains were isolated from the leaf surfaces using the method described by Jiang et al. (2024). The samples were cut into small pieces, immersed in 10 mL of sterilized 0.05% (v/v) Tween 80, shaken for 10 minutes, and the washing solution was serially diluted to 10^–2^. The diluted solution was then spread onto yeast extract–malt extract (YM) agar medium (0.3% yeast extract, 0.3% malt extract, 0.5% peptone, 1% glucose, and 2% agar) supplemented with 200 μg/mL chloramphenicol and incubated at 25 °C for 3 days. Colonies exhibiting distinct yeast morphologies were selected and streaked onto fresh YM agar plates for purification. Following purification, the strains were suspended in YM broth supplemented with 20% (v/v) glycerol and stored at –80 °C for future use.

### Phenotypic characterization

Morphological characteristics, as well as physiological and biochemical properties, were examined using the standard methods described by Kurtzman et al. (2011). Colony and microscopic features were observed on YM agar after 3–7 days of incubation at 25 °C. To assess the inducibility of the sexual state in each isolate, single or mixed strains were incubated on corn meal agar (CMA: 2.5% corn starch and 2% agar), potato dextrose agar (PDA: 20% potato infusion, 2% glucose, and 2% agar), and V8 agar (10% V8 juice and 2% agar) at 20 °C for up to 8 weeks (Li et al. 2020). Ballistoconidium formation was examined using the inverted-plate method (do Carmo-Sousa and Phaff 1962) on CMA at 17 °C. Glucose fermentation was tested in a liquid medium using Durham fermentation tubes. The assimilation of various carbon and nitrogen compounds was tested in liquid media, with starved inocula used for nitrogen testing (Kurtzman et al. 2011). Growth at different temperatures (15, 20, 25, 30, 35, and 37 °C) was evaluated by cultivation on YM agar plates. All novel taxonomic descriptions and proposed names have been deposited in the MycoBank database (http://www.mycobank.org; 8 December 2025). Cultures type are preserved as a metabolically inactive state in the CICC (China Centre of Industrial Culture Collection, Beijing, PR China) and GDMCC (Guangdong Microbial Culture Collection Center, Guangzhou, PR China), cultures ex-type in the PYCC (Portuguese Yeast Culture Collection, Caparica, Portugal).

### DNA extraction, PCR amplification, and sequencing

Genomic DNA was extracted from each strain using the Ezup Column Yeast Genomic DNA Purification Kit, following the manufacturer’s instructions (Sangon Biotech Co., Shanghai, China). The ITS region and the D1/D2 domain of the LSU rRNA were amplified with primers ITS1/ITS4 (White et al. 1990) and NL1/NL4 (Kurtzman and Robnett 1998), respectively. Amplifications were carried out in a 25 µL reaction mixture containing 9.5 µL ddH_2_O, 12.5 µL Taq 2X PCR Master Mix with blue dye (Sangon Biotech Co., Shanghai, China), 1 µL DNA template, and 1 µL of each primer. The PCR protocol was as follows: initial denaturation at 98 °C for 2 minutes, followed by 35 cycles of denaturation at 98 °C for 10 seconds, annealing at 55 °C for 10 seconds, elongation at 72 °C for 15 seconds, and a final elongation at 72 °C for 5 minutes (Chai et al. 2023). The PCR products were then purified and sequenced by Sangon Biotech Co., Ltd (Shanghai, China) using the same primers. The identity and accuracy of each sequence were verified by comparison with sequences in GenBank. Sequence assembly was performed using BioEdit v.7.1.3.0 (Hall 1999). All newly generated sequences have been deposited in the GenBank database (https://www.ncbi.nlm.nih.gov/genbank/).

### Phylogenetic analyses

The sequences obtained in this study, along with reference sequences downloaded from the GenBank database (Table 1), were used for phylogenetic analyses. Individual locus sequences were aligned using MAFFT v.7.110 (Katoh and Standley 2013) and manually refined where necessary using MEGA v.11 (Tamura et al. 2021). Positions that were ambiguous for alignment were excluded using Gblocks v.0.91b (Castresana 2000). Aligned sequences from different loci were concatenated using Phylosuite v.1.2.2 (Zhang et al. 2020). Phylogenetic analyses based on single ITS or D1/D2 sequences were conducted using evolutionary distance data calculated with Kimura’s two-parameter model (Kimura 1980) and the Neighbor-Joining (NJ) method in MEGA v.11 (Tamura et al. 2021). Bootstrap analyses were performed with 1,000 random resamplings. Maximum Likelihood (ML) and Bayesian Inference (BI) analyses based on the combined ITS and D1/D2 sequences were conducted using RAxML v.8.2.3 with 1,000 bootstrap replicates (Stamatakis 2014) and MrBayes v.3.2.7a with 5,000,000 generations (Ronquist et al. 2012), respectively. The best nucleotide substitution model was estimated using Modeltest v.3.04 (Kalyaanamoorthy et al. 2017). The GTR + I + G model was selected for both ML and BI analyses. A bootstrap percentage (BP) above 50% and Bayesian posterior probability (BPPs) above 0.95 were considered as significant support.

**Table 1.** Species name, strain/clone numbers, and GenBank accession numbers included in phylogenetic analyses. Entries in bold represent newly generated materials.

Species name	Strain/clone no.	Location	GenBank accession no.		References
			ITS	LSU D1/D2	
*Carlosrosaea aechmeae*	CBS 14578^T^	Brazil	NR_160562	NG_064406	Felix et al. 2017
*Carlosrosaea betulae*	YN35-7^T^	China	OP470240	OP470144	Li et al. 2020
*Carlosrosaea foliicola*	CGMCC 2.3447^T^	China	NR_174728	MK050282	Jiang et al. 2024
*Carlosrosaea hohenbergiae*	CBS 14563^T^	China	NR_159754	NG_064407	Felix et al. 2017
*Carlosrosaea rhododendri*	JZXS7-21^T^	Brazil	OP470238	OP470142	Jiang et al. 2024
*Carlosrosaea simaoensis*	CGMCC 2.3580^T^	China	NR_174729	MK050283	Li et al. 2020
*Carlosrosaea vrieseae*	UFMG-CM-Y379^T^	Brazil	JX268526	JX280388	Landell et al. 2015
Uncultured fungus	F516_R310	New Zealand	MF976639	–	Unpublished
Uncultured fungus	MFF09GadID	USA	JN890277	–	McGuire et al. 2010
Uncultured fungus	CMH232	USA	KF800323	–	Rittenour et al. 2014
Uncultured fungus	2168_665	Sweden	KP897758	–	Menkis et al. 2015
Uncultured fungus	1978	USA	KX194399	–	Unpublished
Uncultured fungus	3488	USA	KX195909	–	Unpublished
Uncultured fungus	NCD_LSU_otu620	USA	–	KF565590	Unpublished
*Saitozyma carsoniae*	BRIP 72542d^T^	Australia	OP599633	–	Tan and Shivas 2022
*Saitozyma flava*	CBS 331^T^	Japan	NR_073218	NG_057649	Scorzetti et al. 2002
*Saitozyma ninhbinhensis*	JCM 10836^T^	Vietnam	AB261011	AB261011	Golubev 2004
*Saitozyma paraflava*	CBS 10100^T^	China	NR_144774	NG_070557	Golubev 2004
*Saitozyma podzolica*	CBS 6819^T^	–	NR_073213	NG_058283	Scorzetti et al. 2002
*Saitozyma pseudoflava*	CBS 15576^T^	China	MK050284	MK050284	Li et al. 2020
*Saitozyma wallum*	BRIP 66859^T^	Australia	NR_166247	NG_067833	Crous et al. 2019
*Saitozyma* sp.	DBS918-3-4	China	OP470286	OP470190	Unpublished
*Sugitazyma miyagiana*	CBS 7526^T^	Japan	NR_073237	NG_058409	Nakase et al. 1990
*Sugitazyma polliae*	NYNU 24849^T^	China	PQ568990	PQ568988	This study
*Sugitazyma polliae*	NYNU 248167	China	PX630704	PX630706	This study
*Sugitazyma pingtangensis*	NYNU 243166^T^	China	PP837698	PP837697	This study
*Sugitazyma pingtangensis*	NYNU 243228	China	PX630706	PX630705	This study
*Sugitazyma* sp.	PG2-12UV	South Korea	PP373733	–	Unpublished
*Sugitazyma* sp.	YB5-103	South Korea	PP373755	–	Unpublished
*‘Saitozyma*’ sp.	AY564	Germany	–	MK307722	Unpublished
*‘Cryptococcus*’ sp.	D162_2	Bulgaria	–	HM627081	Unpublished
*Sugitazyma* sp.	DBR1-1	USA	–	OP967194	Raudabaugh and Aime 2023
*‘Tremellales*’ sp.	fn_9	Japan	–	LC333466	Tsuji and Fukami 2018
*‘Cryptococcus*’ sp.	GY43L02	Taiwan, China	–	FJ527086	Unpublished
*Tremella diploschistina*	AM199^T^	Papua New Guinea	–	JN790588	Liu et al. 2015
*Tremella diploschistina*	AM200	Papua New Guinea	–	JN790590	Liu et al. 2015
*Tremella parmeliarum*	AM120	Papua New Guinea	–	JN043618	Liu et al. 2015
*Trimorphomyces papilionaceus*	CBS 443.92^T^	Canada	AF444483	AF416645	Oberwinkler and Bandoni 1983
*Trimorphomyces sakaeraticus*	CBS 9934^T^	Thailand	NR_077088	AY211546	Boekhout and Nakase 2011
*Tremella tropica*	CBS 8483^T^	Taiwan, China	NR_155938	NG_058416	Liu et al. 2015

Strains marked with ^“T”^ are ex-type culture.

## Results

### Molecular phylogeny

**Figure 1.**
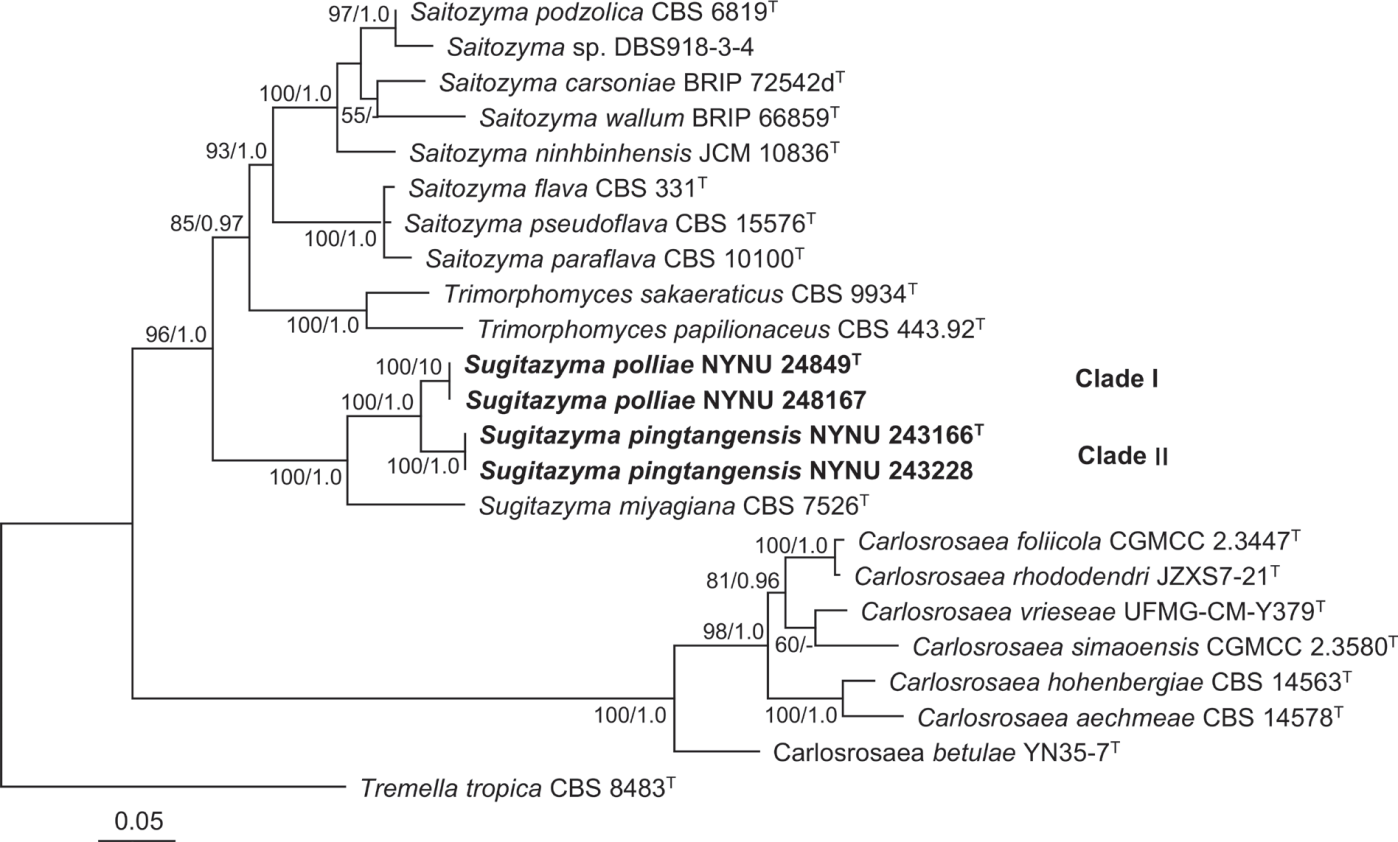
Maximum likelihood (ML) phylogenetic tree of *Sugitazyma* based on combined ITS and D1/D2 sequences. The tree is rooted with *Tremella tropica* CBS 8483. Bootstrap values (BS ≥ 50% and BPP ≥ 0.95) are shown near the branches. Type strain sequences are denoted with **^“T”^**. Newly described species are highlighted in bold.

**Figure 2.**
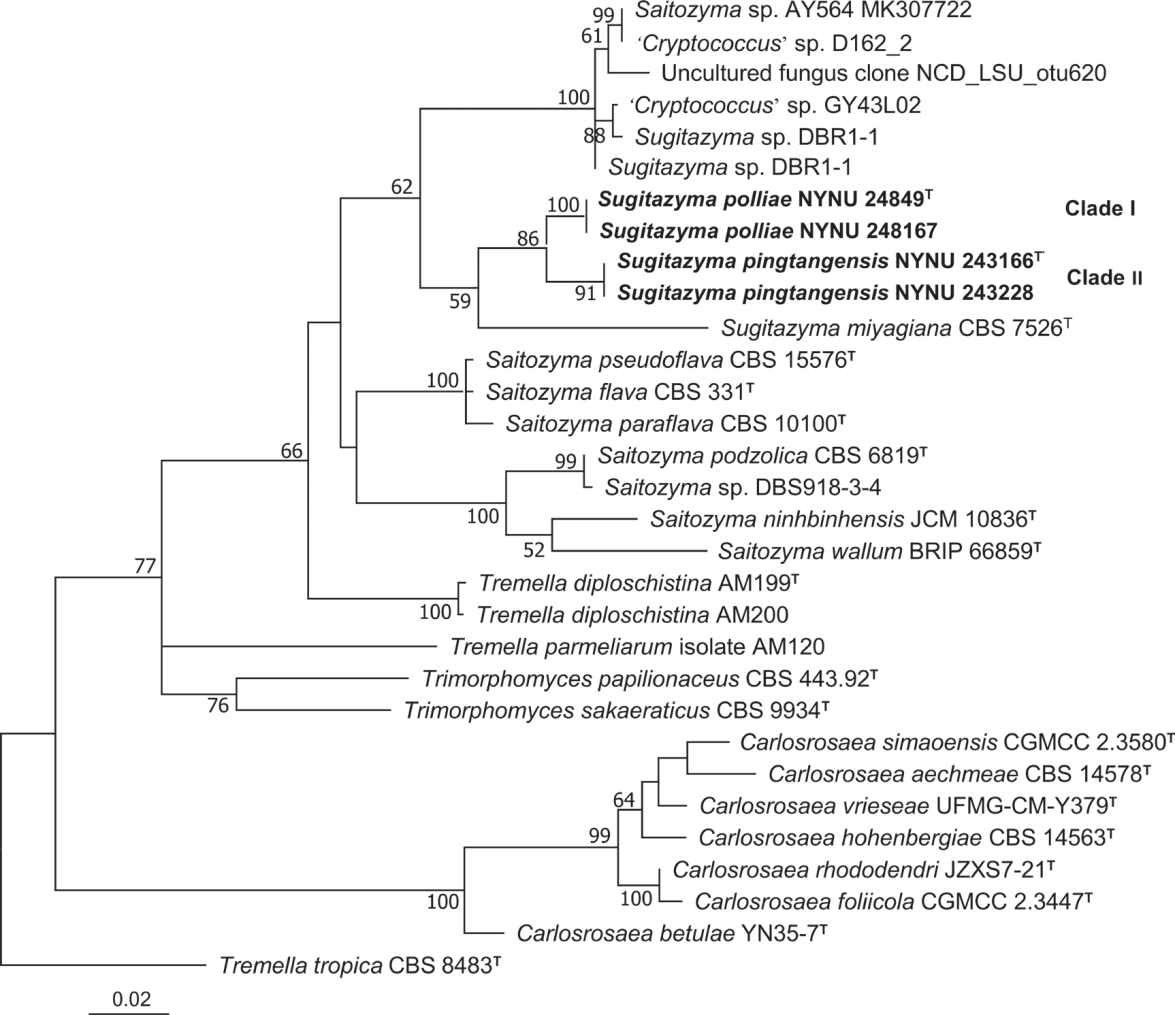
Neighbor-joining (NJ) phylogenetic tree of *Sugitazyma* generated from the D1/D2 sequence data. The tree is rooted with *Tremella tropica* CBS 8483. Bootstrap values (BS ≥ 50%) are shown near the branches. Type strain sequences are denoted with **^“T”^**. Newly described species are highlighted in bold.

**Figure 3.**
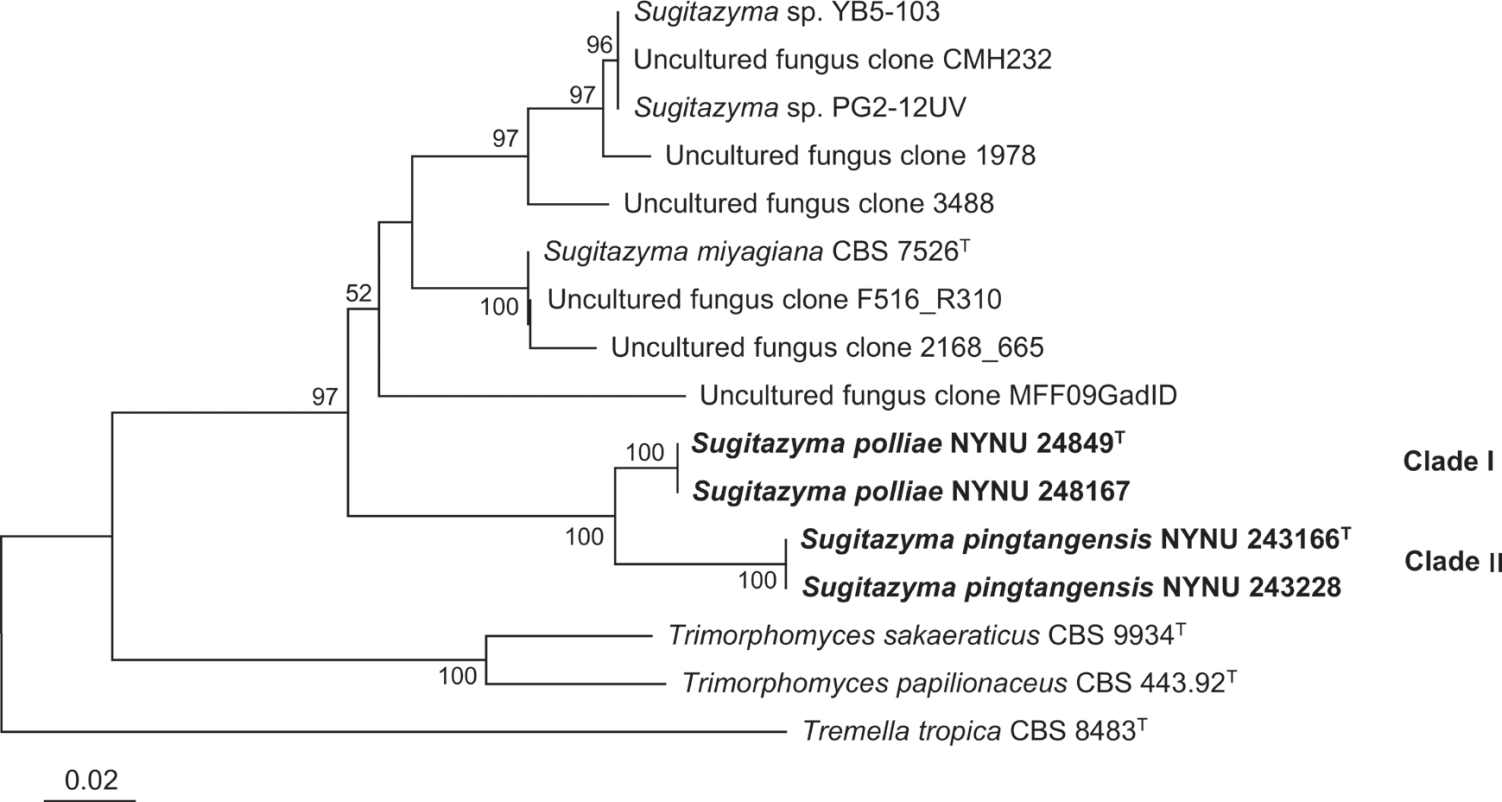
Neighbor-joining (NJ) phylogenetic tree of *Sugitazyma* generated from the ITS sequence data. Bootstrap values (BS ≥ 50%) are shown near the branches. Type strain sequences are denoted with **^“T”^**. Newly described species are highlighted in bold.

The BLAST search tool, based on the ITS and D1/D2 sequences, was used to compare the isolates from this study against the GenBank database. The results showed that the four strains could not be identified as any known yeast species. To determine the phylogenetic placement of the new strains, phylogenetic analyses were performed using both the combined ITS and D1/D2 sequences, as well as single ITS or D1/D2 sequences. The resulting phylogenetic trees revealed that these four strains clustered into two genetically distinct clades, each representing a potential novel species within *Sugitazyma* (Figs 1, 2, 3).

Clade I, comprising strains NYNU 24849 and NYNU 248167, formed a separate branch along with Clade II, which included strains NYNU 243166 and NYNU 243228 (Fig. 1). The strains in Clade I exhibited identical ITS and D1/D2 sequences, indicating conspecificity. Similarly, strains in Clade II also showed identical ITS and D1/D2 sequences but differed from Clade I by 12 nucleotide substitutions (~2.2%) in the D1/D2 domain and 24 nucleotide mismatches (~4.8%) in the ITS region, confirming that they represent two distinct species. These two clades are closely related to *S. miyagiana* in trees based on the combined ITS and D1/D2 sequences, as well as single D1/D2 sequences (Figs 1, 2). However, when single ITS sequences were analyzed, the two clades clustered at the base of the *Sugitazyma* clade (Fig. 3). Pairwise comparisons revealed that these two clades differ from both previously described and undescribed *Sugitazyma* species by more than 25 nucleotide substitutions (~4.5%) in the D1/D2 domain and over 20 nucleotide mismatches (~4.6%) in the ITS region. Based on these phylogenetic results, we propose that the two clades represent two novel species within the genus *Sugitazyma*, which are named *Sugitazyma polliae* sp. nov. and *Sugitazyma pingtangensis* sp. nov.

### Taxonomy

#### Sugitazyma polliae

Taxon classificationFungiTremellalesTrimorphomycetaceae

C.Y. Chai & F.L. Hui sp. nov.

82FE0ABE-BBFD-5D67-8BE6-F8F9DEB3D52C

861585

Fig. 4

##### Etymology.

The specific epithet polliae refers to *Pollia*, the plant genus from which the type strain was isolated.

##### Typus.

China • **Hainan**: Wuzhishan City; Wuzhi Mountain; on the phylloplane of *Pollia japonica*; Aug 2024; S.L. Lv leg., NYNU 24849 (holotype GDMCC 2527^T^ preserved as a metabolically inactive state, metabolically inactive ex-type culture PYCC 10137, GenBank numbers: ITS-PQ568990, D1/D2-PQ568988).

**Figure 4.**
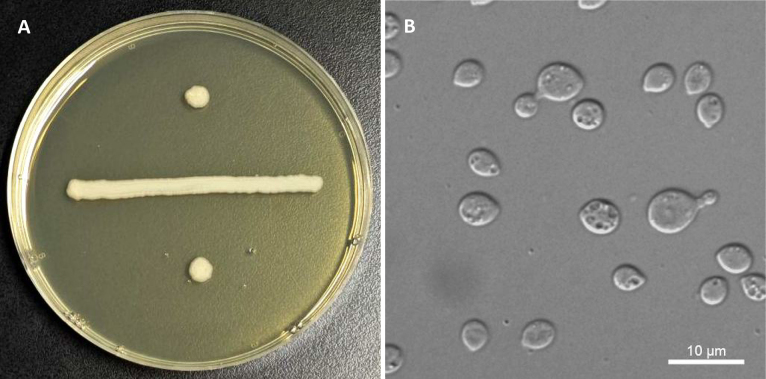
*Sugitazyma polliae* (NYNU 24849). **A**. Colony on YM agar after 7 days at 20 °C; **B**. Budding cells on YM agar after 3 days at 20 °C.

##### Description.

On YM agar after 7 days at 20 °C, the streak culture cream, mucoid and smooth, with entire margin. After 3 days on YM agar at 20 °C, single cells ovoid, 5–6 × 6.5–13 μm, polar budding, on denticles or short stalks, with percurrent proliferation. After 1 month at 20 °C, a pellicle and a sediment are present. In Dalmau plate culture on CMA, pseudohyphae are not formed. Sexual structures are not observed in any of the strains or when strains are paired on PDA, CMA or V8 agar. On corn meal agar, ballistoconidia are not produced. Glucose fermentation is absent. Glucose, inulin (weak), sucrose (weak), raffinose (weak and delayed), melibiose (weak and delayed), galactose (weak), lactose (weak), trehalose (weak), maltose (weak), melezitose (weak), methyl-α-D-glucoside, cellobiose (weak), salicin (weak), D-xylose (weak), L-arabinose (weak), D-arabinose, 5-keto-D-gluconate, D-ribose (weak and delayed), ethanol, erythritol (delayed), ribitol (delayed), galactitol (weak and delayed), D-mannitol (weak and delayed), D-glucitol, myo-inositol (weak), DL-lactate (weak), succinate, citrate (delayed), D-gluconate (weak), 2-keto-D-gluconate (weak), D-glucuronate (weak), and glucono-1,5-lactone are assimilated as sole carbon sources. L-Sorbose, L-rhamnose, methanol, glycerol, D-glucosamine, and N-acetyl-D-glucosamine are not assimilated. Nitrate, nitrite, ethylamine (delayed), and L-lysine are assimilated as sole nitrogen sources. Cadaverine is not assimilated. Maximum growth temperature is 25 °C. Growth on 50% (w/w) glucose-yeast extract agar is negative. There is hydrolysis of urea and starch formation. The DBB reaction is positive.

##### Additional strain examined.

China • **Hainan**: Wuzhishan City; Wuzhi Mountain; on the phylloplane of *Symplocos adenophylla*; Aug 2024; S.L. Lv leg., NYNU 248167; GenBank numbers: ITS-PX630704, D1/D2-PX630706.

##### Note.

Physiologically, *S. polliae* differs from its closely related species, *S. pingtangensis*, described in this study, by its inability to assimilate L-sorbose and L-rhamnose, as well as its ability to assimilate myo-inositol. *S. polliae* also differs from *S. miyagiana* in its inability to assimilate L-rhamnose, its growth capacity at 30 °C, and its ability to assimilate inulin, galactitol, and D-mannitol (Table 2).

**Table 2.** Physiological and biochemical characteristics of *Sugitazyma* species.

Characteristics	*S. polliae*	*S. pingtangensis*	S. miyagiana*
Carbon assimilation			
Inulin	w	d, w	–
L-Sorbose	–	d	–
L-Rhamnose	–	d, w	+
Galactitol	d, w	+	–
D-Mannitol	d, w	+	–
Myo-inositol	w	–	+
Nitrogen assimilation			
Nitrate	+	+	–
Nitrite	+	+	n
Growth tests			
Growth at 30 °C	–	–	+

Note. + positive reaction; – negative reaction; d, delayed positive; w, weakly positive. All data from this study, except* which were obtained from the original description (Boekhout et al. 2011).

#### Sugitazyma pingtangensis

Taxon classificationFungiTremellalesTrimorphomycetaceae

C.Y. Chai & F.L. Hui sp. nov.

0282AF78-99B7-5442-9A1B-A37CC0282C52

861586

Fig. 5

##### Etymology.

The specific epithet pingtangensis refers to the geographic origin of the type strain, Pingtang County, Guizhou.

##### Typus.

China • **Guizhou**: Pingtang County; on the phylloplane of *Millettia pachycarpa*; Mar 2024; D. Lu, leg., NYNU 243166 (holotype CICC 33642^T^ preserved as a metabolically inactive state, metabolically inactive ex-type culture PYCC 10058, GenBank numbers: ITS-PP837698, D1/D2-PP837697).

##### Description.

On YM agar after 7 days at 20 °C, the streak culture is cream, tough and rough, with an entire margin. After 3 days on YM agar at 20 °C, single cells globose to ovoid, 6–8.5 × 6.5–12 μm, polar budding, on denticles or short stalks, with percurrent proliferation. After 1 month at 20 °C, a ring and a sediment are present. In Dalmau plate culture on CMA, pseudohyphae are not formed. Sexual structures are not observed in any of the strains or when strains are paired on PDA, CMA or V8 agar. On corn meal agar, ballistoconidia are not produced. Glucose fermentation is absent. Glucose, inulin (weak and delayed), sucrose, raffinose (weak and delayed), melibiose (weak and delayed), galactose, lactose, trehalose, maltose, melezitose, methyl-α-D-glucoside, cellobiose, salicin, L-sorbose (delayed), L-rhamnose (weak and delayed), D-xylose, L-arabinose, D-arabinose, 5-keto-D-gluconate, D-ribose (delayed), ethanol (weak), erythritol (weak and delayed), ribitol, galactitol, D-mannitol, D-glucitol, DL-lactate (weak), succinate, citrate, D-gluconate, 2-keto-D-gluconate, D-glucuronate, and glucono-1,5-lactone are assimilated as sole carbon sources. Methanol, glycerol, myo-inositol, D-glucosamine, and N-acetyl-D-glucosamine are not assimilated. Nitrate, nitrite, ethylamine (delayed), and L-lysine are assimilated as sole nitrogen sources. Cadaverine is not assimilated. Maximum growth temperature is 25 °C. Growth on 50% (w/w) glucose-yeast extract agar is negative. There is hydrolysis of urea and starch formation. The DBB reaction is positive.

**Figure 5.**
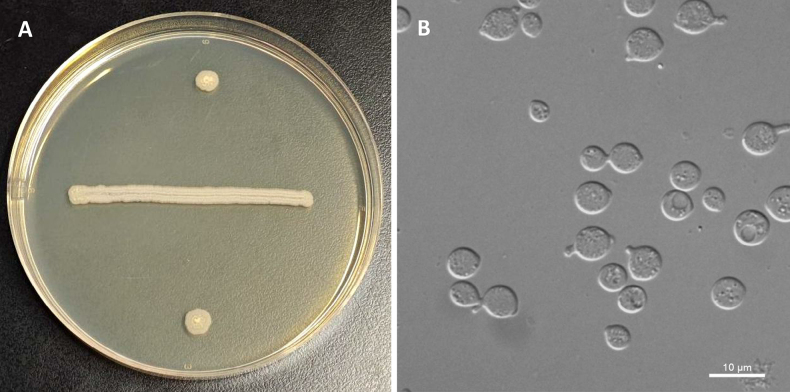
*Sugitazyma pingtangensis* (NYNU 243166). **A**. Colony on YM agar after 7 days at 20 °C; **B**. Budding cells on YM agar after 3 days at 20 °C.

##### Additional strain examined.

China • **Guizhou**: Pingtang County; on the phylloplane of *Millettia pachycarpa*; Mar 2024; D. Lu leg., NYNU 243228; GenBank numbers: ITS-PX630706, D1/D2-PX630705.

##### Note.

Physiologically, *S. pingtangensis* differs from *S. polliae*, by its inability to assimilate myo-inositol and its ability to assimilate L-sorbose and L-rhamnose; and from *S. miyagiana* in its inability to assimilate myo-inositol, its growth capacity at 30 °C, and its ability to assimilate inulin, L-sorbose, galactitol, D-mannitol, and nitrate (Table 2).

### Key to the species in *Sugitazyma*

**Table:** 

1	Inulin is not assimilated	***S. miyagiana***
–	Inulin is assimilated	**2**
2	L-Rhamnose is assimilated	***S. pingtangensis***
–	L-Rhamnose is not assimilated	***S. polliae***

## Discussion

In this study, *S. polliae* and *S. pingtangensis* are proposed as two novel species of the genus *Sugitazyma* based on phylogenetic evidence and phenotypic characteristics. Accordingly, the number of formally recognized species in *Sugitazyma* is increased from one to three. In addition, phylogenetic analyses based on single ITS or D1/D2 sequences revealed seven undescribed or erroneously identified strains and seven uncultured fungal clones may represent more than nine species in *Sugitazyma* clade (Figs 2, 3), which need to be clarified in the future because their ITS or D1/D2 sequences are not available at present. In our opinion, this is the first time both single and two-locus analyses is used in such a phylogenetic and taxonomic study on *Sugitazyma*, which can provide the basis for future taxonomy and phylogenetic study of the genus.

Members of the genus *Sugitazyma* have not been sufficiently studied, and the diversity within this genus remains poorly understood. To date, only three *Sugitazyma* species, including the two described in this study, have been identified in nature. *S. miyagiana*, the type species of the genus, was originally isolated from the surface of *Abies firma* collected in Japan (Nakase et al. 1990). *S. polliae* and *S. pingtangensis*, introduced in the current study, were isolated from healthy leaves collected in tropical and subtropical regions of China. However, several unpublished or erroneously identified strains have been isolated from different parts of the world. For example, ‘*Saitozyma*’ sp. AY564 (MK307722) was obtained from Germany, ‘*Cryptococcus*’ sp. D162_2 (HM627081) from Bulgaria, *Sugitazyma* sp. DBR1-1 (OP967194) from the USA (Raudabaugh and Aime 2023), ‘*Tremellales*’ sp. fn_9 (LC333466) from Japan (Tsuji and Fukami 2018), and ‘*Cryptococcus*’ sp. GY43L02 (FJ527086) from Taiwan, China. Additionally, *Sugitazyma* sp. PG2-12UV (PP373733) and *Sugitazyma* sp. YB5-103 (PP373755) were isolated from South Korea (Figs 2, 3). Several uncultured fungal clones, such as clone NCD_LSU_otu620 (KF565590) from soil in the USA, clone OTU_F516_R310 (MF976639) from living leaves in New Zealand, clone MFF09GadID (JN890277) from forest soil in the USA (McGuire et al. 2010), clone CMH232 (KF800323) from house dust in the USA (Rittenour et al. 2014), clone 2168_665 (KP897758) from *Picea abies* needles in Sweden (Menkis et al. 2015), and clones 1978 (KX194399) and 3488 (KX195909) from soil in the USA, also belong to *Sugitazyma* based on the analyses of single ITS or D1/D2 sequences (Figs 2, 3). These findings suggest that this genus may be widely distributed across diverse environments, and further large-scale studies are required to explore the diversity and distribution of *Sugitazyma* species globally. Ultimately, these studies will significantly enhance our understanding of the diversity, distribution, and ecology of *Sugitazyma*.

## Supplementary Material

XML Treatment for Sugitazyma polliae

XML Treatment for Sugitazyma pingtangensis

## Additional information

### Use of AI

No use of AI was reported.

### Author ORCIDs

Peng Wang https://orcid.org/0009-0001-8289-9640

Chun-Yue Chai https://orcid.org/0000-0001-7753-6223

Qiu-Hong Niu https://orcid.org/0000-0003-1695-7117

Feng-Li Hui https://orcid.org/0000-0001-7928-3055

### Data availability

All of the data that support the findings of this study are available in the main text.
